# Grand Challenges in Surgical Oncology

**DOI:** 10.3389/fonc.2012.00127

**Published:** 2012-10-02

**Authors:** Umberto Veronesi, Vaia Stafyla

**Affiliations:** ^1^European Institute of OncologyMilan, Italy

Surgical oncology is a field of Medicine that presented a rapid evolution over the past decades. The increasing incidence of malignancies throughout the globe and their detrimental effects led to an increasing interest in discovering new curative techniques, that could affect and prolong the patients’ life. Surgery has been the cornerstone and its course underwent dramatic changes over the years. Since the beginning of the twentieth century, the aim of the surgeons was to offer the maximal tolerable treatment, as their efforts were focused on delivering as extensive surgical treatments as the patients could tolerate, in order to achieve an acceptable disease control. This approach resulted in unnecessary mutilating interventions that had tremendous effects on the patients’ quality of life. The most characteristic example of this era was the radical mastectomy performed for breast cancer, that was a devastating experience for breast cancer survivors. At the end of the 1960s, when it was made evident that the prognosis was mainly linked to the presence of distant metastases and not to the extent of the local treatment, the opposite trend was developed. The main objective was to identify the minimal effective treatment, targeting at the preservation of the anatomy and functionality of the affected organ, that would also improve the patients’ quality of life. At the same time emerged the necessity for a new set of investigations for a better control of occult metastases.

The advances in the field of medical oncology have led to an improved control of the systemic disease. Adjuvant chemotherapy and endocrine therapy became an important component of the treatment, aiming at the improvement of overall survival in breast, colon, and lung cancer. More recently the new biomolecular targeted treatments pointed at increasing the therapeutic efficiency, sparing the oncological patient the debilitating side effects of the traditional chemotherapy. Her 2 inhibitors for breast cancer, imatinib for GIST, BRAF inhibitors for melanoma, EGFR tyrosine kinase inhibitors for non-small cell lung cancer are just some of the examples of targeted therapies that recently became standard treatments.

The value of radiotherapy is well known, as it is often essential for obtaining local control (Stea et al., 2011). The progression of technology changed the delivery of radiation from two-dimensional radiotherapy to three-dimensional conformal radiotherapy and recently to intensity modulated radiation therapy. Image guided radiotherapy, stereotactic body radiotherapy, and stereotactic radiosurgery are some other modern methods that emerged over the past decade (Orvieto et al., 2011). Brachytherapy and intraoperative radiotherapy are new promising modalities that allow the delivery of radiotherapy for either short periods or during the surgical intervention, thus minimizing the extend of radiation fields and concentrating the major part of therapeutic intervention in only one session, aiming at improving the patients quality of life.

Neoadjuvant treatments are currently being used for downstaging. Chemotherapy and radiotherapy, as well as hormone therapy for hormone dependent malignancies, in the pre-operative setting are shown to decrease the extent of the solid tumors, rendering them operable, without excising the affected organ.

Similarly, the progress of Nuclear Medicine has improved the results of surgical oncology in the diagnostic setting, with the technique of the sentinel lymph node, used in breast cancer, in melanoma, in gynecological, and head and neck tumors. Positive emission tomography is becoming an essential tool for whole body staging of cancer. In the therapeutic setting, radioactive iodine ablation is used for thyroid malignancies and recently radioimmunotherapy with yttrium seems to have promising results not only for hematologic malignancies, but also for solid tumors (Pohlman et al., 2006).

The latest generations of surgeons have witnessed the transition from open surgery to laparoscopic or video-assisted surgery and soon after to robotic surgery. This is all attributed to the revolution of technology, that allows the surgeon to access the cavities of the human body and operate inside them using sophisticated instruments, that make extremely precise and delicate movements under the surgeon’s commands. Laparoscopic surgery is widely used in the last 20 years for malignancies of all intra and extraperitoneal organs, while video-assisted surgery is a mainstay in thoracic surgery. Robotic surgery is already established in many fields, especially in urology, with a large number of radical prostatectomies, as well as nephrectomies, performed robotically. The main benefit of all these minimally invasive techniques is the limited insult, not only of the target organ, but also of the adjacent structures, resulting in a faster recovery and a shorter hospitalization, maintaining in most cases the normal physiological functions.

It is clear that the implementation of the above mentioned approach can only be performed in cases of early, small sized, cancers confined to the target organ. Early detection of cancer has contributed significantly to the advance of surgical oncology, as it was shown to affect both the treatment strategy toward minimal intervention and organ saving techniques, and the prognosis. The population screening programs are an essential part of every national health system and their beneficial effect is well known. The mammography for breast cancer, the colonoscopy for colon cancer, the pap-test for cervical cancer, and the spiral computed tomography for lung cancer are included in the screening guidelines of all health organizations and had a noteworthy impact on these malignancies.

Despite the enthusiasm of all the progress that has been achieved in the field of technology, which has altered many of the traditional concepts in surgical oncology in the last 50 years, the big challenge that the modern surgical oncologist is facing is the integration of all the new knowledge in a system that will be patient centered. Multidisciplinary approach for cancer cases and decision making are basic elements for the management of the oncologic patient. The surgical oncologist should be part of a multidisciplinary team that discusses every single cancer case and aims at selecting the best available combination of diagnostic and therapeutic procedures, taking into consideration the different characteristics of each patient. All cancer centers worldwide have already implemented this team approach in oncology, as the available modalities for diagnosis and treatment are continuously increasing and each specialist is becoming an expert in a certain field. The goal of the multidisciplinary meeting is to decide the most suitable approach, tailored to the needs of every patient, using the most appropriate procedures of every relevant field of medicine. Inter-professional communication, patient centered medical approach, and integrated health delivery are new concepts that should characterize the surgical oncology of the future. The surgical oncologist should not remain isolated in the operating theater, but he/she should learn to interact with other specialists and develop non-technical skills, like communication, leadership, organization, and situational awareness (Aggrawal and Grantcharov 2011).

The role of training and continuous education in surgical oncology is fundamental in the era of information technology and super-specialization. The surgical oncologist should be exposed to a large number of operations and should become familiar with advanced technology instruments and techniques. At the same time he/she should not only act as a technician, but as a real scientist, being able to understand the results of basic and clinical research beyond the surgical domain, and integrate them into his/her everyday practice. Information technology is a useful tool for disseminating on line knowledge in every part of the world and thanks to it, continuous education is made feasible.

Probably the most important challenge in the surgical oncology of the twenty-first century remains the philosophic value of humanism. In the era of rapid technological advances, of functional genomics and proteomics applied to cancer, of increasing health market, the doctor-patient relationship should be based on trust, respect toward the doctor and on selflessness, availability, honesty, and compassion toward the patient. The surgical oncologist is required to act in his/her patient’s interest even if it may conflict his/her own and his main task is not just to prolong the patient’s life, but to improve its quality. He/she should approach the patients not as cancer cases, aiming at treating the cancer, but he/she should take into to account the human parameters of each patient, aiming at curing the cancer patient of his/her disease, and its psychological consequences.
